# The Effect of Stigma on Family Planning and HIV Pre-exposure Prophylaxis Decisions of Young Women Accessing Post-Abortion Care in Kenya

**DOI:** 10.1007/s10461-024-04274-6

**Published:** 2024-03-07

**Authors:** Yasaman Zia, Lydia Etyang, Margaret Mwangi, Roy Njiru, Felix Mogaka, Lavender June, Irene Njeru, Job Makoyo, Susan Kimani, Kenneth Ngure, Inviolata Wanyama, Elizabeth Bukusi, Bernard Nyerere, Cyprian Nyamwaro, Nelly Mugo, Renee Heffron, PrEDIRA team, Sue Peacock, Kathy Thomas, Josephine Odoyo, Florence Mwangi

**Affiliations:** 1 Department of Epidemiology, University of Washington, Seattle, WA USA; 2 Department of Global Health, University of Washington, Seattle, WA USA; 3 Partners in Health and Research Development, Kenya Medical Research Institute, Nairobi, Kenya; 4 Research Care and Training Program, Kenya Medical Research Institute, Thika, Kenya; 5 Marie Stopes Kenya, Nairobi, Kenya; 6 School of Public Health, Jomo Kenyatta University of Agriculture and Technology, Nairobi, Kenya; 7 Department of Medicine, University of Alabama at Birmingham, 845 19th Street South / BBRB 256, Birmingham, AL 35294-2170 USA

**Keywords:** PrEP, Abortion, Stigma, Adolescent girls and young women

## Abstract

Adolescent girls and young women (AGYW) in Eastern and Southern Africa face parallel epidemics of unintended pregnancy and HIV. Their sexual health decisions are often dominated by intersecting stigmas. In an implementation science project integrating delivery of daily, oral pre-exposure prophylaxis (PrEP) for HIV prevention into 14 post-abortion care (PAC) clinics in Kenya, we enrolled a subset of PrEP initiating AGYW (aged 15 to 30 years) into a research cohort. Utilizing log binomial models, we estimated the effect of PrEP stigma on PrEP continuation (measured via self-report and urine assay for tenofovir) and abortion stigma on contraceptive initiation. Between April 2022 and February 2023, 401 AGYW were enrolled after initiating PrEP through their PAC provider, of which 120 (29.9%) initiated highly-effective contraception. Overall, abortion and PrEP stigmas were high in this cohort. Abortion stigma was more prevalent among those that were adolescents, unmarried, and reported social harm. Among 114 AGYW returning for the month 1 follow-up visit, 83.5% reported continuing PrEP and 52.5% had tenofovir detected. In this subset, higher levels of PrEP stigma were significantly associated with greater likelihood of PrEP adherence, but not PrEP continuation. For abortion stigma, greater scores in the subdomain of isolation were significantly associated with greater likelihood of initiating a highly-effective contraception, while greater scores in the subdomain of community condemnation were significantly associated with reduced likelihood of initiating a highly-effective contraception. Given the burden of stigma documented by our work, PAC settings are a pivotal space to integrate stigma-informed counseling and to empower young women to optimize contraceptive and PrEP decisions.

**Supplementary Information:**

The online version contains supplementary material available at 10.1007/s10461-024-04274-6.

## Plain English Summary

Stigma perpetuates social and health inequities by limiting individual’s acceptance of and access to essential care. In particular, young women experience a heavy burden of stigma that influences their sexual health choices. Our goal was to estimate the effect of stigma on reproductive health decisions among young women in abortion settings in Kenya. We enrolled into research a cohort of young women in Kenya who had recently received postabortal care and had chosen to start taking PrEP. In our study, 120 (29.9%) of women initiated a highly-effective contraceptive method, the most common methods being oral contraceptives (11.0%) or injectable (10.7%). At month 1 follow-up, 95 (78.5%) women reported continuing PrEP and 60 (50.0%) were adherent, as detected via urine testing. We found that abortion stigma influenced initiation of a family planning method after an abortion, stemming from feelings of isolation as well as community condemnation. We also found that PrEP stigma was common and higher among those who were adherent to PrEP. In conclusion, stigma is a common experience for young women to navigate when making sexual health decisions. These findings highlight the need for interventions at multiple levels to address stigma and optimize health decision-making among young women so that they prevent unintended pregnancy and HIV and reduce stigma-driven inequities that pose a critical barrier to sexual and reproductive health care.

## Background

While there have been pronounced efforts to increase rollout and uptake of HIV pre-exposure prophylaxis (PrEP), adolescent girls and young women (AGYW) continue to bear the burden, experiencing 63% of new HIV infections in the African region [1]. AGYW in Eastern and Southern Africa represent a disproportionate percentage of new HIV infections. In Kenya, AGYW have a 2.2-fold higher HIV incidence than their male age counterparts and represent one-third of all new infections yearly [2, 3]. HIV prevention programs aimed at AGYW cite high PrEP uptake but frequent discontinuation, with approximately 50–85% of AGYW discontinuing PrEP within 1–3 months [4–8]. This discontinuation from PrEP has been interpreted to signify an incongruence of daily pill-taking with AGYW lifestyle, including the inconvenience, unfamiliarity, and stigma with daily pill-taking [9, 10].

In addition to HIV, AGYW in Kenya face an epidemic of unintended pregnancy [11, 12]. More than 40% of unintended pregnancies end with an abortion, and AGYW comprise nearly half of all clients seeking post-abortion care (PAC) [11, 12]. While abortion is currently illegal in Kenya except in cases where a person’s life or health is in danger, people experiencing or recovering from abortion (whether spontaneous or not) can access PAC. PAC settings provide a set of core interventions for essential reproductive health care, including emergency treatment for incomplete abortions, provision of family planning, and sexual health counseling [13, 14]. For AGYW receiving PAC, risk for subsequent pregnancy is high for certain age groups, given previous findings contraceptive uptake among Kenyan AGYW accessing PAC has been estimated as 6% for those aged 10–18, 21% for those aged 19–21, and 63% for those aged 22–26, and may be coupled by risk for HIV and other sexually transmitted infections [15, 16]. Thus, PAC is an underutilized setting for the integration of PrEP and is likely to serve an unmet need for HIV prevention services for AGYW.

The combination of factors, or a syndemic of psychosocial and health conditions, that may exacerbate HIV and unintended pregnancy risk among AGYW include factors such as social status, relationship dynamics, and healthcare access [17, 18]. The context in which AGYW make PrEP decisions is often dominated by intersecting considerations about stigma around HIV medications, sexual activity, and youth and female autonomy [19–25]. AGYW often face difficult challenges navigating their sexual autonomy given age-specific vulnerabilities such as a lack of agency or experience in negotiating sex, heightened awareness of social stigma pertaining to gender norms and AGYW’s societal status, economic disempowerment sometimes leading to transactional sex, and difficulty accessing sexual health information and services [26–32]. The defining feature of stigma includes a degree of bias or unfairness that denies individuals full social acceptance and thereby drives social and health inequities [33, 34]. The complexity of stigma experiences of AGYW in PAC settings is an insufficiently studied area of research and is an important consideration of sexual health decision-making and the adoption of services that further provides autonomy and safety.

In this implementation science project delivering PrEP services within PAC clinics in Kenya, we hypothesized that stigma has a bearing on women’s sexual and reproductive health decision-making processes, and we conducted analyses to estimate the effect of (1) PrEP stigma on PrEP adherence and continuation and (2) abortion stigma on family planning initiation among AGYW.

## Methods

PrEDIRA II is an implementation science project to deliver PrEP integrated into PAC services provided at 14 clinics in Kisumu, Nairobi, and Thika, Kenya. Prior to launch, technical advisors (TAs) conducted facility-based trainings for the rollout of the PrEP program and facilitated linkages between each facility and Ministry of Health supply chain for PrEP commodities. Facility-based trainings included PAC clinic-specific training on HIV risk assessment and clinical management of PrEP clients and technical support, including technical advice, quality improvement, and program strengthening, to clinic staff to deliver PrEP. During the program, TAs provided support to facility staff who counseled and prescribed PrEP, and abstracted data on PrEP dispensing from medical charts. Eligibility for PrEP included an HIV-negative test, a clinical assessment with no medical contraindications, and self-reported sexual activity, in accordance with Kenya national guidelines [35]. Women initiating PrEP through PAC continued to receive PrEP care, including HIV testing and PrEP refills, through the PAC program or another PrEP location of their choice.

A subset of AGYW initiating PrEP through PrEDIRA II were enrolled into a research cohort to evaluate the factors that shaped PrEP care over time. All PAC clients who were initiating PrEP were offered enrollment into a 6-month research cohort, and the first 400 to be interested and eligible were enrolled (94.4% acceptance rate, n = 425 offered). In addition to PrEP initiation through a participating PAC, eligibility in the research cohort was limited to women between the ages 15–30 years who were willing and able to provide written informed consent. Research procedures included visits one month after PrEP initiation and quarterly thereafter up to 6 months. At each visit, participants completed interviewer-administered questionnaires to assess demographics, sexual behavior and partnership characteristics, empowerment, social harm (emotional, physical, or economic harm by their partner), mental health, social support, HIV risk perceptions and PrEP readiness, anticipated PrEP disclosure, family planning methods, PrEP use, and sexually transmitted infection symptoms. Urine was collected for tenofovir (TFV) measurement at all follow up visits. To meet the objectives of the larger study, all PAC clinics were randomized to provide either the foundational PrEP program or an enhanced PrEP program that included multiple post-PrEP check-in calls and appointment reminder cards.

### HIV Prevention Stigma Scale (HPSS)

The HIV Prevention Stigma Scale (HPSS) was recently developed among men who have sex with men (MSM) in the US [36]. This unidimensional measure includes 2 parts (Likert and Semantic Differential), captures anticipated, experienced, and internalized stigma, and has not yet been validated outside of the US context, but has been used among MSM in South Africa [37] and serodiscordant couples in Mozambique [38]. We utilized the 13 questions from the Likert portion of HPSS, which is measured on a range of 1 to 5, with each point increase indicating higher stigma. The HPSS was collected among PrEDIRA II clients at enrollment. Through consultation with team leadership, technical advisors, and younger members of the PrEDIRA team, we systematically reviewed each of the questions, made adaptations based on the study context, and piloted revised questions with the study team. For example, for the HPSS item around people using PrEP being viewed as “slutty,” we also added the word “loose” to reflect cultural connotations around promiscuity (Supplementary Materials).

### Individual Level Abortion Stigma (ILAS)

The Individual Level Abortion Stigma (ILAS) scale [39] is a widely-used continuous measure that has 20 items and captures four subdomains: worries about judgement, isolation, self-judgement, and community condemnation. The scale has a range of 1 to 5, with each point increase indicating higher stigma. The internal consistency and reliability have been assessed among young women in Kenya [40]. We adapted the ILAS scale items to initiate questions with wording to include both pregnancy loss and abortion to capture the variety of pregnancy experiences women in PAC settings may face (Supplementary Material).

### PrEP Use

Self-reported PrEP continuation was assessed during the Month 1 follow-up visit by asking if the participant is using PrEP. PrEP continuation was measured by quantifying tenofovir (TFV) in a point-of-care urine assay (Abbott/Alere Rapid Diagnostics) [41] 1-month after initiating PrEP and quarterly thereafter. Results were used by program staff to provide feedback to AGYW in real time about TFV detection and to engage AGYW in informed counseling about PrEP continuation and potential HIV exposure. The test was performed by trained personnel after self-collection of urine into sterile cups by research participants. TFV detection corresponds to PrEP use the past 3 days [41].

### Contraceptive Initiation

Contraceptive use was assessed at enrollment, by asking what methods of contraceptive the participant initiated subsequent to their abortion services. We categorized highly-effective contraception as use of an injectable, implantable device, intrauterine device, or oral contraceptives. The referent category included those who reported use of condoms or no contraceptives.

### Statistical Methods

We used descriptive statistics to characterize the demographics, partnership dynamics, and both stigma measures (HPSS and ILAS) among young women included in this cohort. To assess the internal consistency of each scale, we calculated the Cronbach’s alpha. To create a dichotomous categorization of each stigma scale, we used the 75th percentile as the cut point to indicate high stigma used in each model.

We conducted log-binomial regression to estimate the relative risk of high PrEP stigma with demographic and partnership factors. To assess the relationship between PrEP stigma and PrEP adherence and continuation at Month 1, we used separate log binomial regression models. A priori, we decided to include the type of PAC clinic (private or public) that the client attended in these regression models [42]. For models that included PrEP adherence and continuation, we also included clinic-level enhanced arm assignment a priori. For models with PrEP adherence and continuation as the outcomes, we conducted three analyses to account for participants who did not attend their Month 1 visit and to assess the possible range of effects in our existing data: (1) excluded participants without a Month 1 visit, (2) assigned all participants who did not attend as non-adherent and discontinuing PrEP, and (3) assigned all participants who did not attend as adherent and continuing PrEP.

We conducted log-binomial regression to estimate the relative risk of high abortion stigma with demographic and partnership factors. To assess the relationship between abortion stigma and highly-effective contraceptive initiation, we used log binomial regression models. We decided a priori to include the type of PAC clinic (private or public) that the client attended and reported experiences of social harm [42, 43]. All analyses were conducted in SAS version 9.4 (Cary, NC).

## Results

Between April 2022 and February 2023, a total of 401 AGYW between the ages of 15-and 30-year initiating PrEP were enrolled into research. The median age was 22 years (interquartile range (IQR): 20–25), 40.9% were currently partnered, and 60.3% earned an income in the past year (Table 1). In terms of partnership characteristics, some women had new sexual partners in the past 3 months (18.5%), and most did not know their partners’ HIV status(es) (61.5%). After receiving PAC services, 120 (29.9%) women initiated a highly-effective contraceptive method (10.7% injectable, 11.0% oral). Among 30% (n = 114) of women who attended their month 1 follow-up, 95 (83.5%) women reported continuing PrEP and 60 (52.5%) had urine TFV detected.

**Table 1:** Baseline and follow-up characteristics of AGYW in PrEDIRA II

	n/N or Mean (SD)	% or Median (IQR)
*Baseline*		
Age		
15–19	73/401	18.3%
20–24	203/401	50.8%
25–30	124/401	31.0%
Marital status		
Single	111/401	28.0%
Partnered	162/401	40.9%
Married	123/401	31.1%
Income earned in past 12 m	240 /401	60.3%
Partner provides financial support	71/401	18.0%
New partner in past 3 months	74/401	18.5%
Recent sexual partners living with HIV		
Yes	4/401	1.0%
No	150/401	37.5%
Don’t know	246/401	61.5%
Sex in the past 3 months	364/401	90.8%
Condom usage in past 3 months		
Never	283/363	78.0%
Rarely	27/363	7.4%
Sometimes	51/363	14.1%
Often	2/363	0.6%
Type of family planning initiated		
Injectable	43/401	10.7%
Implant	31/401	7.7%
IUD	2/401	0.6%
Oral contraceptive	44/401	11.0%
Condoms	8/401	2.0%
None	274/401	68.3%
Highly-effective contraception use	120/401	29.9%
Overall HPSS score	2.83 (0.56)	2.92 (2.46—3.23)
High PrEP stigma	117/401	29.8%
*ILAS domain*		
Overall score	2.5 (0.55)	2.6 (2.1–2.9)
Worries about judgement	1.9 (0.99)	1.6 (1.0–2.7)
Isolation	2.4 (1.09)	2.5 (1.2 – 3.3)
Self-Judgement	2.8 (0.79)	2.5 (2.6–3.0)
Community condemnation	4.5 (0.7)	5.0 (4.0–5.0)
High abortion stigma	93/401	23.2%
*M1 follow-up*		
Self-reported PrEP continuation	95/114	83.5%
TFV detected in POC urine assay	60/114	52.5%

*HPSS* HIV Prevention Stigma Scale, *ILAS* Individual-Level Abortion Stigma scale, *IQR* interquartile range, *IUD* intrauterine device, *POC* point-of-care, *SD* standard deviation, *TFV* tenofovir

### PrEP Stigma

**Fig. 1:**
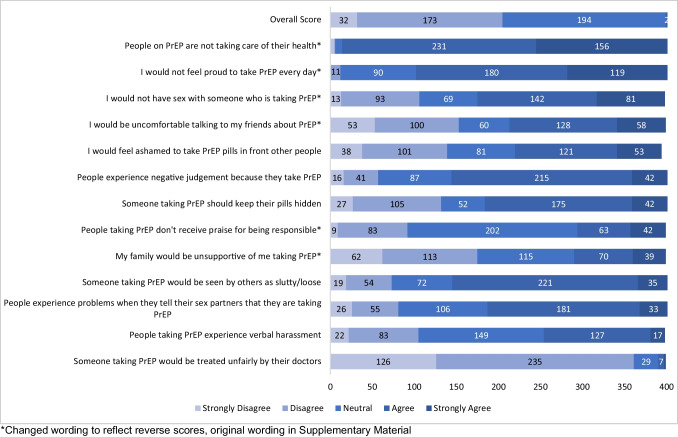
Results of Adapted HPSS Likert Scale (5-point scale)

Overall, participants had scores that indicated high PrEP stigma on the HPSS scale. Items that most frequently had a response of “Strongly agree” or “Agree,” indicating a high level of stigma, included people taking PrEP “are not taking care of their health,” “would be viewed as ‘slutty’ or ‘loose,’” “experiencing negative judgement,” “would not feel proud to take PrEP daily,” “would experience problems telling their sexual partner,” and would not be someone they’d have sex with (Fig. 1). The item most often with “Strongly disagree” or “Disagree” was “someone taking PrEP would be treated unfairly by their doctor.” The Likert portion of the HPSS scale had a Cronbach’s alpha of 0.81, suggesting internal consistency. There were no significant demographic or partnership characteristics correlated with high PrEP stigma (Table 2).

**Table 2:** Demographic and Partnership Factors Associated with High Stigma among AGYW in PrEDIRA 2 research cohort

	Baseline high PrEP stigma (N = 401)		Baseline high abortion stigma (N = 400)	
	N (%)	RR (95% CI)	N (%)	RR (95% CI)
*Baseline demographics*				
Age group				
15–19	25 (34.3%)	1.33 (0.86, 2.05)	27 (37.0%)	1.99 (1.24, 3.21)
20–24	59 (29.1%)	1.13 (0.78, 1.63)	42 (20.8%)	1.12 (0.71, 1.78)
25–30	32 (25.8%)	REF	23 (18.6%)	REF
Relationship Status				
Single w/no partner	28 (25.2%)	0.87 (0.59, 1.27)	34 (26.1%)	2.09 (1.26, 3.49)
Partnered	31 (19.1%)	0.74 (0.52, 1.06)	35 (21.7%)	1.7 (1.03, 2.81)
Married	33 (26.8%)	REF	17 (13.8%)	REF
Income earned in past 12 months				
Yes	70 (29.2%)	0.98 (0.72, 1.34)	54 (22.6%)	1.01 (0.59, 1.74)
No	47 (29.8%)	REF	38 (24.1%)	REF
New sexual partner in past 3 months				
Yes	21 (38.9%)	1.25 (0.86, 1.82)	12 (22.2%)	1.12 (0.54, 2.31)
No	94 (31.1%)	REF	66 (21.9%)	REF
Recent sexual partners living with HIV				
Yes	0 (0%)	*	–	–
No	47 (35.1%)	REF	–	–
Don’t know	68 (31.2%)	0.89 (0.66, 1.2)	–	–
Condom usage in past 3 months				
Never or rarely	93 (30.0%)	REF	78 (25.2%)	REF
Sometimes or often	19 (35.9%)	1.3 (0.71, 2.4)	9 (17.0%)	0.61 (0.28, 1.3)
Social Harm in past 3 months				
Any Social Harm	15 (22.4%)	0.73 (0.46, 1.18)	22 (32.8%)	1.56 (1.05, 2.33)
None	102 (30.5%)	REF	70 (21.0%)	REF

*Model did not converge due to insufficient data

Among a subset who attended Month 1 visit, PrEP stigma was significantly associated with PrEP adherence when measured via urine TFV (adjusted relative risk (aRR): 1.33, 95% CI: 1.07, 1.66; Table 3), after adjustment for clinic type and enhanced/standard of care PrEP program type. However, PrEP stigma was not significantly associated with self-reported PrEP continuation (aRR: 1.08, 95% CI 0.99, 1.18), after adjusting for only clinic type due to convergence. When missing data were assigned PrEP outcomes of non-adherent and discontinuing, the effects of stigma on PrEP adherence and PrEP continuation were closer to the null and non-significant. When the missing data were assigned to outcomes of adherent and continuing, PrEP stigma and PrEP continuation were also closer to the null and the association with adherence was statistically significant (aRR: 1.07, 95% CI: 1.01, 1.14).

**Table 3:** Association between baseline PrEP stigma and PrEP continuation among AGYW in the PrEDIRA 2 Research Cohort, by women with M1 follow-up visit and overall

	Low Stigma N (%)	High Stigma N (%)	Overall N (%)	RR (95% CI)	aRR* (95% CI)
**Subset with Month 1 Visit attended (N = 114)**					
PrEP continuation at month 1 via TFV detection					
PrEP Stigma Score	41 (45.6%)	19 (63.3%)	60 (50.0%)	1.41 (1.15, 1.73)	1.33 (1.07, 1.66)
PrEP continuation at month 1 via self-report					
PrEP Stigma Score	69 (75.8%)	26 (86.7%)	95 (78.5%)	1.11 (1.02, 1.22)	1.08** (0.995, 1.18)
**Scenario 1: With PrEP outcomes assigned for those with missing data as non-adherent and discontinuing (N = 401)**					
PrEP continuation at month 1 via TFV detection					
PrEP Stigma Score	41 (14.4%)	19 (16.2%)	60 (15.0%)	1.11 (0.73, 1.7)	1.4 (0.91, 2.15)
PrEP continuation at month 1 via self-report					
PrEP Stigma Score	69 (24.3%)	26 (22.2%)	95 (23.7%)	0.86 (0.64, 1.15)	0.99 (0.73, 1.35)
**Scenario 2: With PrEP outcomes assigned for those with missing data as adherent and continuing (N = 401)**					
PrEP continuation at month 1 via TFV detection					
PrEP Stigma Score	235 (82.8%)	106 (90.6%)	341 (85.0%)	1.09 (1.06, 1.14)	1.07 (1.01, 1.14)
PrEP continuation at month 1 via self-report					
PrEP Stigma Score	262 (92.3%)	113 (95.6%)	375 (93.5%)	1.04 (1.01, 1.06)	1.03 (0.97, 1.09)

*Adjusted for enhanced arm of PrEDIRA enhanced activities and whether the clinic was public or private

**Adjusted for whether the clinic was public or private, due to convergence

### Abortion Stigma

At enrollment, respondent’s ILAS scores were distributed towards the middle of the scale (mean: 2.53, standard deviation (SD): 0.55, Table 1). Responses to the self-judgment subdomain (mean: 2.88, SD 0.79) and community condemnation subdomain (mean: 4.45, SD 0.7) were concentrated on the high end of the scale while responses to the worries about judgement subdomain (mean: 1.9, SD 0.99) were the concentrated on the low end of the scale. The ILAS scale had Cronbach’s alpha score of 0.81, suggesting internal consistency.

High abortion stigma was significantly more common among clients that were single (26.1% vs. 13.1%, relative risk (RR): 2.09, 95% CI: 1.26–3.49, Table 2) and partnered (21.7% vs. 13.1%, RR: 1.7, 95% CI: 1.03, 2.81) as compared to women who were married. High abortion stigma was significantly more common among clients that were aged 15–19 years (37.0% vs. 18.6%, RR: 1.99, 95% CI: 1.24, 3.21) as compared to women who were 25–30 years. High abortion stigma was also significantly more common among AGYW who reported experiences of social harm by any of their partners in the past 3 months (32.8% vs. 21.0%, RR: 1.56, 95% CI: 1.05, 2.33) as compared to those who did not report experiences of social harm.

Participants with higher abortion stigma scores were significantly more likely to initiate highly-effective contraception (adjusted RR (aRR): 1.48, 95% CI: 1.19, 1.84; Table 4) after adjustment for reported experiences of social harm and whether the clinic the client attended was private or public. The subdomain of isolation (aRR: 1.59, 95% CI: 1.38, 1.84) was significantly associated with an increase in highly-effective contraception use, while the subdomain of community condemnation (aRR: 0.83, 95% CI: 0.72, 0.96) was significantly associated with a reduction in highly-effective contraception use.

**Table 4:** Association between Abortion Stigma and Initiation of Highly-Effective Contraception among AGYW in PrEDIRA 2 Research Cohort

	Initiated contraception Low Stigma N (%)	Initiated contraception High Stigma N (%)	RR (95% CI)	aRR* (95% CI)
Overall Score	87 (28.3%)	33 (35.5%)	1.46 (1.18–1.81)	1.48 (1.19 – 1.84)
*Subdomains*				
Worries about judgement	90 (30.4%)	30 (28.7%)	0.95 (0.81–1.1)	0.94 (0.81 – 1.1)
Isolation	63 (21.7%)	57 (51.4%)	1.59 (1.37–1.83)	1.59 (1.38–1.84)
Self Judgement	52 (21.8%)	68 (42.0%)	1.14 (0.96—1.35)	1.15 (0.96–1.36)
Community Condemnation	85 (43.8%)	35 (16.9%)	0.83 (0.72–0.96)	0.83 (0.72–0.96)

*Adjusted for experiences of social harm and whether the clinic was private or public

## Discussion

At the intersection of HIV prevention and abortion care, this is the first study, to our knowledge, to assess the overlapping stigmas associated with each that are experienced by young women and their effects on reproductive health decisions. The syndemic of psychosocial conditions that affect young women accessing PrEP and abortion services in Kenya present challenges to sexual and reproductive health access, quality, and wellbeing. When examining the subdomain and item scores of ILAS and HPSS, we found a high burden of both abortion stigma and PrEP stigma among young women in this study. Among a subset of women who attended their Month 1 visit, higher PrEP stigma scores were observed among women who were adherent to PrEP when measured via laboratory assay, but not among women who reported continuation of PrEP. Higher abortion stigma scores were detected among women who initiated highly-effective contraception, and we note that women initiating PrEP and not intiating PrEP may have different experiences with both abortion stigma and contraception. Collectively, these findings highlight the levels of stigma that are present among young women being offered PrEP and contraception in PAC settings as well as the complexity of roles that stigma may play in sexual health decision-making.

Our results were surprising in light of the direction of the association: higher stigma preceded greater PrEP continuation and coincided with greater frequency of contraceptive uptake. One potential reason is that these prevention methods enable AGYW to avoid outcomes from which stigma manifests, namely becoming infected with HIV or needing an abortion. Given that stigma drives health inequities and impedes scalability of prevention programs in communities, multilevel interventions to address community, provider, and internalized stigma, such as PrEP education dissemination at each of these levels, are needed to improve access and experiences with needed services [33].

In other studies, abortion stigma has negatively influenced sexual and reproductive health outcomes through pathways that lead to isolation, secrecy, and unawareness of safe abortion methods [44]. Considering the role of contraception in preventing unintended pregnancy, PAC settings can be optimal to deliver contraception and counseling to reduce the burden of abortion stigma. In this study, high abortion stigma was more prevalent among those that were adolescents (aged 15–19), unmarried, and reported social harm, pointing to subgroups that may benefit from counseling to reduce stigma. Only 30% of women across 14 PAC clinics initiated highly-effective contraception after their abortion, which may be motivated by fears of side effects and misconceptions around some contraceptives [45]. The subdomain of community condemnation (mean: 4.5 out of 5) was a nearly universal experience of abortion stigma reported by young women, and was the latent construct driving down the overall association between abortion stigma and initiation of highly-effective contraception. Similar findings have been reported among secondary-school students in Kenya, in that abortion is widely viewed as sin (89% of respondents) and as shameful for the person’s family (73%) [46]. Alternatively, the subdomain of isolation, which aligns with being unable to disclose their abortion to and feeling unsupported by people they were close to, was associated with increases in highly-effective contraception use. This finding suggests that discretion may be operating as a protective factor to receive abortion care that is highly stigmatized. These subdomains indicate that the decision to use highly-effective contraception was related to different aspects of stigma, stemming from the community and social isolation. Qualitative work has previously described abortion stigma among women in Kenya, and women receiving PAC have described self-reliance as being vital to overcome internalized and perceived stigma, isolation and secrecy as necessities to avoid stigma, and challenges accessing and affording safe abortion services [47–52]. Together, these findings highlight the importance of identifying opportunities to reduce stigma and diminish its effect on young women.

Few validated measures of PrEP stigma exist and are tailored for communities in Africa, and so consequently the impact of stigma has been insufficiently captured as a determinant of engagement in HIV prevention. Qualitative studies have outlined how stigma shapes and is interwoven into AGYW’s decisions to use and disclose their use of PrEP to their sexual partners and family members [10]. In settings similar to our study, AGYW have cited that PrEP stigma can derive from HIV-related misconceptions around HIV medications and promoting sexual promiscuity [19]. We observed that PrEP stigma was associated with a 30% higher PrEP adherence, aligning with other studies that found persistent stigma among AGYW users of PrEP [10, 53–55]. However, this finding is likely an upper estimate of the effect since our sample was affected by high loss to follow up and we saw lower estimates in our models with imputed data that were reflective of PrEP discontinuation among participants who did not attend their follow-up visit.

We contextualize our recommendations within the myriad of social conditions of young women that require attention, including poverty, access to care, experiences of intimate partner violence, and sociopolitical harms, and that limit women’s ability to achieve sexual health wellness [56]. Given the intersectional nature of stigma, engaging AGYW in care requires multilevel interventions including, but not limited to, community- and clinic-level interventions eliminating stigma from sexual and reproductive health counseling and individual-level interventions to garner informed decision making [57]. As found in other studies, eradicating intersectional stigma by combating misconceptions around PrEP and/or contraceptives, utilizing strengths-based approaches tailored for young people, and promoting informed decision-making shifts the motivations for PrEP and contraceptive use to AGYW and can improve retention in care [58, 59]. In addition, provider bias has been found as factor limiting PrEP prescription to marginalized groups, thereby driving disparities within those with an acute need for sexual health counseling and services [60]. However, aligning motivations for providing care with their hesitations toward PrEP and contraceptive provision may overcome these challenges [61]. To meet AGYW’s coverage needs for PrEP and/or contraceptives, overcoming stigma surrounding essential healthcare requires both fostering open communication with providers and placing young women’s psychosocial needs at the center of programs and interventions.

Some limitations of this work include the lack of a scale to measure PrEP stigma that was culturally adapted to Kenyan AGYW. We were not able to conduct cognitive interviews to assess potential participants’ understanding of the HPSS. While no scales developed for young women initiating PrEP existed at the time of this project launch, other scales of PrEP stigma for priority populations in Kenya have since become available and can be used in future work to validate our findings [62]. Selection bias may be underlying the findings of PrEP stigma and its effect on increased PrEP adherence and continuation in this study. When imputing data for 70% of young women who did not return to their Month 1 visit, our point estimates moved closer to the null and some statistical significance was lost. Additionally, some women may have experienced a loss of a desired pregnancy, and their choices around contraceptive methods may reflect these desires. Some women who did not return to their Month 1 visit could potentially have received PrEP care elsewhere, and we did not account for this in any way. Lastly, social desireability and white-coat bias may underlie PrEP continuation outcomes when measured either through self-report or biological measure.

## Conclusions

Building on previous implementation projects of PrEP for AGYW, evidence suggests that raising the quality of care and improving uptake of contraception and continuation of HIV prevention methods require counseling that addresses overlapping stigmas and their impact on health decision-making. As suggested by previous research, young women face conflicting messaging around their sexual health decisions from multiple angles, surrounding them with societal, familial, and partner-level expectations of their choices to have or delay sex, when to prevent or carry a pregnancy, to avoid HIV, but not take medications, and to use condoms or not [26–32]. The moral imperative that is placed on young women’s sexuality is rooted in intersectional stigma, which further limits their acceptability of and access to essential sexual health services [58]. Together, these themes demonstrate the importance of normalizing sexual health services such as PrEP care, abortion care, and contraceptive choices.

Post-abortion care settings could be an optimal space to integrate stigma-informed counseling to empower young women to choose care that aligns with their needs, as evidenced by the burden of both PrEP and abortion stigma observed in our study. In parallel, the role of values-clarification to address provider’s hesitations to prescribe PrEP to young women is also indispensable in delivering unbiased information and fostering patient-centered care as supported by other studies [63]. In summary, our findings highlight much-needed multilevel interventions to address stigma and optimize health decision-making in order to provide young women with the tools needed to avoid unintended pregnancy and HIV and to address stigma-driven inequities as a critical barrier to the provision of sexual and reproductive health care.

### Supplementary Information

Below is the link to the electronic supplementary material.

Supplementary file1 (DOCX 17 KB)

## Data Availability

Data are available upon request from icrc@uw.edu.
