# Supplementary material for: The Effect of Stigma on Family Planning and HIV Pre-exposure Prophylaxis Decisions of Young Women Accessing Post-Abortion Care in Kenya
Source: AIDS Behav. 2024 Mar 7;28(6):1834–44. doi: 10.1007/s10461-024-04274-6 (PMC11161434; doi:10.1007/s10461-024-04274-6)
Supplement: Supplementary file 1 — Supplementary file1 (DOCX 17 KB) [file 10461_2024_4274_MOESM1_ESM.docx]

**Supplementary Material**

| 1. I would feel ashamed to take PrEP pills in front other people 2. Someone taking PrEP should keep their pills hidden 3. People experience negative judgement because they take PrEP 4. I would have sex with someone who is taking PrEP* 5. Someone taking PrEP would be seen by others as slutty**/loose** 6. People taking PrEP receive praise for being responsible* 7. ~~My friends would be supportive of me taking PrEP*~~ **I would be comfortable talking to my friends about PrEP*** 8. Someone taking PrEP would be treated unfairly by their doctors 9. People experience problems when they tell their sex partners that they are taking PrEP 10. I would feel proud to take PrEP every day* 11. People taking PrEP experience verbal harassment 12. People on PrEP are taking care of their health* 13. My family would be supportive of me taking PrEP* | 1-Strongly disagree  2- Disagree  3- Neutral  4- Agree  5- Strongly agree |
| --- | --- |

HPSS Likert Scale Revisions

ILAS Scale Revisions

| Worries about judgement | Around the time of my abortion, I was worried…  1. Other people might find out about my abortion 2. My abortion would negatively affect my relationship with someone I love 3. I would disappoint someone I love 4. I would be humiliated 5. People would gossip about me 6. I would be rejected by someone I love 7. People would judge me negatively | 1- Not worried  2- A little worried  3- Quite worried  4- Extremely worried |
| --- | --- | --- |
| Isolation | Around the time of or since my **pregnancy loss or** abortion…  8. I have had a conversation with someone I am close with about my abortion* 9. I was open with someone that I am close with about my feelings about my abortion* 10. I felt the support of someone that I am close with at the time of my abortion*  11. I can talk to the people ~~I am~~ close ~~with~~ **to me** about my abortion* 12. I can trust the people ~~I am~~ close **to me** with information about my abortion* 13. When I had my abortion, I felt supported by the people I was close with* | 1- Never  2- Once  3- More than once  4- Many times |
| Self Judgement | Around the time of my **pregnancy loss or** abortion, I felt…  14. I felt ~~like a bad person~~ **relieved** * 15. I felt confident I had made the right decision* 16. I felt ashamed about my abortion 17. I felt selfish 18. I felt guilty | 1- Strongly disagree  2- Disagree  3- Neutral  4- Agree  5- Strongly agree |
| Community Condemnation | **We want to explore community perceptions when a woman chooses to terminate a pregnancy.** How many people in your community held the following beliefs?  19. **Self-induced pregnancy loss or** abortion is always wrong  20. **Self-induced pregnancy loss or** abortion is the same as murder | 1- No one  2- A few people  3- About half of people  4- Many people  5- Most people |
